# Supplementary material for: Lysosomal Membrane Stability of Mussel (Mytilus galloprovincialis Lamarck, 1819) as a Biomarker of Cellular Stress for Environmental Contamination
Source: Toxics. 2023 Jul 26;11(8):649. doi: 10.3390/toxics11080649 (PMC10459067; doi:10.3390/toxics11080649)
Supplement: Supplementary file 1 [file toxics-11-00649-s001.zip › toxics-2507731-supplementary.pdf]

toxics

MDPI

*Supplementary Materials*

**Table S1.** Seawater concentration of organic contaminants. Data are expressed as mean values. PAHs: polycyclic aromatic hydrocarbons; OCPs: organochlorine pesticides; PCBs: polychlorinated biphenyls; Σ: sum of concentrations of organic contaminant compounds; *lod*: limit of detection. MDP: Midia Port; MPB: Mamaia Pescarie Bay; CTP: Constanta Port; MGP: Mangalia Port.

| Contaminant | Sampling site | | | |
|---|---|---|---|---|
| | **MDP** | **MPB** | **CTP** | **MGP** |
| | ***PAHs (µg/L)*** | | | |
| Naphthalene | 0.0043 | 0.0001[lod] | 0.8969 | 0.0048 |
| Acenaphthylene | 0.0001[lod] | 0.0001[lod] | 0.0001[lod] | 0.0001[lod] |
| Acenaphthene | 0.0001[lod] | 0.0001[lod] | 0.0001[lod] | 0.0001[lod] |
| Fluorene | 0.0004 | 0.0001[lod] | 0.0242 | 0.0002 |
| Phenanthrene | 0.0001[lod] | 0.0019 | 4.3762 | 0.0001[lod] |
| Anthracene | 0.0001[lod] | 0.0010 | 1.1510 | 0.0001[lod] |
| Fluoranthene | 0.0001[lod] | 0.0001[lod] | 0.0107 | 0.0001[lod] |
| Pyrene | 0.0001[lod] | 0.0001[lod] | 0.0338 | 0.0001[lod] |
| Benzo[a]anthracene | 0.0001[lod] | 0.0001[lod] | 0.0001[lod] | 0.0001[lod] |
| Chrysene | 0.0001[lod] | 0.0001[lod] | 0.0001[lod] | 0.0001[lod] |
| Benzo[b]fluoranthene | 0.0001[lod] | 0.0001[lod] | 0.0001[lod] | 0.0001[lod] |
| Benzo[k]fluoranthene | 0.0001[lod] | 0.0001[lod] | 0.0001[lod] | 0.0001[lod] |
| Benzo[a]pyrene | 0.0001[lod] | 0.0001[lod] | 0.0001[lod] | 0.0001[lod] |
| Benzo[g,h,i]perylene | 0.0001[lod] | 0.0001[lod] | 0.0001[lod] | 0.0001[lod] |
| Dibenzo[a,h]anthracene | 0.0001[lod] | 0.0001[lod] | 0.0001[lod] | 0.0001[lod] |
| Indeno [1,2,3-c,d]pyrene | 0.0001[lod] | 0.0001[lod] | 0.0001[lod] | 0.0001[lod] |
| ∑PAHs | 0.0062 | 0.0043 | 6.4939 | 0.0064 |
| TPHs | 1235.417 | 2.000 | 30.583 | 43.291 |
| | ***OCPs (µg/L)*** | | | |
| HCB | 0.004[lod] | 0.004[lod] | 0.004[lod] | 0.004[lod] |
| Lindane | 3.221 | 3.513 | 11.739 | 4.414 |
| Heptachlor | 0.003[lod] | 8.356 | 0.003[lod] | 0.003[lod] |
| Aldrin | 7.175 | 9.029 | 7.628 | 10.044 |
| Dieldrin | 1.846 | 6.645 | 0.002[lod] | 2.722 |
| Endrin | 0.884 | 2.219 | 0.341 | 1.346 |
| p,p'DDE | 4.123 | 5.109 | 4.605 | 4.131 |
| p,p'DDD | 17.700 | 19.858 | 0.002[lod] | 76.130 |
| p,p'DDT | 0.002[lod] | 9.939 | 0.002[lod] | 0.002[lod] |
| ∑OCPs | 34.957 | 64.672 | 24.326 | 98.795 |
| | ***PCBs (µg/L)*** | | | |
| PCB 28 | 0.004[lod] | 0.004[lod] | 0.004[lod] | 0.004[lod] |
| PCB 52 | 0.006[lod] | 0.006[lod] | 0.006[lod] | 0.006[lod] |
| PCB 101 | 0.006[lod] | 0.006[lod] | 0.164 | 0.006[lod] |
| PCB 118 | 0.004[lod] | 0.004[lod] | 0.004[lod] | 0.004[lod] |
| PCB 138 | 0.007[lod] | 0.009 | 0.007[lod] | 0.007[lod] |
| PCB 153 | 0.009[lod] | 0.009[lod] | 0.009[lod] | 0.009[lod] |
| PCB 180 | 0.003[lod] | 0.003[lod] | 0.003[lod] | 0.003[lod] |
| ∑PCBs | 0.039 | 0.041 | 0.200 | 0.039 |

**Table S2.** Seawater concentration of heavy metals. Data are expressed as mean values. ΣHM: sum of concentrations of heavy metal compounds. MDP: Midia Port; MPB: Mamaia Pescarie Bay; CTP: Constanta Port; MGP: Mangalia Port.

| Contaminant | Sampling site | | | |
|---|---|---|---|---|
| | **MDP** | **MPB** | **CTP** | **MGP** |
| ***Heavy metals (µg/L)*** | | | | |
| Copper (Cu) | 3.690 | 14.710 | 4.670 | 5.870 |
| Cadmium (Cd) | 0.011 | 0.006 | 0.002 | 0.017 |
| Lead (Pb) | 0.988 | 0.581 | 0.543 | 2.757 |
| Nickel (Ni) | 1.120 | 0.780 | 1.060 | 1.100 |
| Chromium (Cr) | 12.450 | 13.990 | 15.780 | 10.880 |
| ∑HMs | 18.259 | 30.067 | 22.055 | 20.624 |

**Table S3.** *Mytilus galloprovincialis* tissue concentration of organic contaminants. Data are expressed as mean values. *dw*: dry weight; *lod*: detection limit. PAHs: polycyclic aromatic hydrocarbons; OCPs: organochlorine pesticides; PCBs: polychlorinated biphenyls; Σ: sum of concentrations of organic contaminant compounds. MDP: Midia Port; MPB: Mamaia Pescarie Bay; CTP: Constanta Port; MGP: Mangalia Port.

| Contaminant | Sampling site | | | |
|---|---|---|---|---|
| | **MDP** | **MPB** | **CTP** | **MGP** |
| ***PAHs (µg/kg dw)*** | | | | |
| Naphthalene | 0.042 | 0.143 | 0.088 | $0.100^{lod}$ |
| Acenaphthylene | $0.100^{lod}$ | $0.100^{lod}$ | $0.100^{lod}$ | $0.100^{lod}$ |
| Acenaphthene | $0.100^{lod}$ | $0.100^{lod}$ | $0.100^{lod}$ | $0.100^{lod}$ |
| Fluorene | $0.100^{lod}$ | $0.100^{lod}$ | $0.100^{lod}$ | $0.100^{lod}$ |
| Phenanthrene | 0.389 | 0.704 | 0.577 | 0.068 |
| Anthracene | 0.406 | 0.115 | 0.141 | $0.100^{lod}$ |
| Fluoranthene | 0.010 | $0.100^{lod}$ | $0.100^{lod}$ | $0.100^{lod}$ |
| Pyrene | 0.028 | $0.100^{lod}$ | $0.100^{lod}$ | $0.100^{lod}$ |
| Benzo[a]anthracene | $0.100^{lod}$ | $0.100^{lod}$ | $0.100^{lod}$ | $0.100^{lod}$ |
| Chrysene | 0.010 | $0.100^{lod}$ | $0.100^{lod}$ | $0.100^{lod}$ |
| Benzo[b]fluoranthene | $0.100^{lod}$ | $0.100^{lod}$ | $0.100^{lod}$ | $0.100^{lod}$ |
| Benzo[k]fluoranthene | $0.100^{lod}$ | $0.100^{lod}$ | $0.100^{lod}$ | $0.100^{lod}$ |
| Benzo[a]pyrene | $0.100^{lod}$ | $0.100^{lod}$ | $0.100^{lod}$ | $0.100^{lod}$ |
| Benzo[g,h,i]perylene | $0.100^{lod}$ | $0.100^{lod}$ | $0.100^{lod}$ | $0.100^{lod}$ |
| Dibenzo[a,h]anthracene | $0.100^{lod}$ | $0.100^{lod}$ | $0.100^{lod}$ | $0.100^{lod}$ |
| Indeno[1,2,3-c,d]pyrene | $0.100^{lod}$ | $0.100^{lod}$ | $0.100^{lod}$ | $0.100^{lod}$ |
| ∑PAHs | 1.885 | 2.261 | 2.106 | 1.568 |
| ***OCPs (µg/kg dw)*** | | | | |
| HCB | $0.500^{lod}$ | $0.500^{lod}$ | $0.500^{lod}$ | $0.500^{lod}$ |
| Lindane | 845.238 | $0.400^{lod}$ | $0.400^{lod}$ | $0.400^{lod}$ |
| Heptachlor | 3090.303 | 2591.325 | 2124.739 | 3451.341 |
| Aldrin | $0.300^{lod}$ | $0.300^{lod}$ | $0.300^{lod}$ | $0.300^{lod}$ |
| Dieldrin | $0.300^{lod}$ | $0.300^{lod}$ | $0.300^{lod}$ | $0.300^{lod}$ |
| Endrin | 15.153 | $0.400^{lod}$ | $0.400^{lod}$ | $0.400^{lod}$ |
| p,p'-DDE | $0.200^{lod}$ | $0.200^{lod}$ | $0.200^{lod}$ | $0.200^{lod}$ |
| p,p'-DDD | $0.200^{lod}$ | $0.200^{lod}$ | $0.200^{lod}$ | $0.200^{lod}$ |
| p,p'-DDT | $0.200^{lod}$ | $0.200^{lod}$ | $0.200^{lod}$ | $0.200^{lod}$ |
| ∑OCPs | 3952.394 | 2593.825 | 2127.239 | 3453.841 |
| ***PCBs (µg/kg dw)*** | | | | |

| Contaminant | Sampling site | | | |
|---|---|---|---|---|
| | MDP | MPB | CTP | MGP |
| PCB 28 | 0.400[lod] | 0.400[lod] | 0.400[lod] | 0.400[lod] |
| PCB 52 | 7301.648 | 6688.920 | 4734.084 | 6945.534 |
| PCB 101 | 98.909 | 149.932 | 0.600 | 59.510 |
| PCB 118 | 1026.580 | 1127.532 | 717.735 | 1066.248 |
| PCB 138 | 0.700[lod] | 0.700[lod] | 0.700[lod] | 0.700[lod] |
| PCB 153 | 0.600[lod] | 0.600[lod] | 0.600[lod] | 0.600[lod] |
| PCB 180 | 21.624 | 31.618 | 100.811 | 85.956 |
| ∑PCBs | 8450.461 | 7999.703 | 5554.930 | 8158.948 |

**Table S4.** *Mytilus galloprovincialis* tissue concentration of heavy metals. Data are expressed as mean values. *dw*: dry weight. ΣHM: sum of concentrations of heavy metal compounds. MDP: Midia Port; MPB: Mamaia Pescarie Bay; CTP: Constanta Port; MGP: Mangalia Port.

| Contaminant | Sampling site | | | |
|---|---|---|---|---|
| | MDP | MPB | CTP | MGP |
| *Heavy metals (μg/kg dw)* | | | | |
| Copper (Cu) | 4017.0 | 6599.0 | 4514 | 2998.0 |
| Cadmium (Cd) | 835.7 | 1331.0 | 662.5 | 350.4 |
| Lead (Pb) | 1880.0 | 2755.0 | 1944.0 | 481.6 |
| Nickel (Ni) | 974.5 | 2712.0 | 1023.0 | 879.1 |
| Chromium (Cr) | 559.0 | 747.7.0 | 1341.0 | 626.7 |
| Cobalt (Co) | 133.5 | 305.0 | 214.8 | 35.0 |
| ∑HMs | 8399.7 | 14449.7 | 9699.3 | 5370.8 |

**Table S5.** Kruskal–Wallis non-parametric test (two-tailed) and post-hoc test (Dunn's test for pair-wise comparisons) ($n$ = 15). *K1*: observed value; *K2*: critical value; *df*: degree of freedom; *p*: *p*-value (one-tailed). MDP: Midia Port; MPB: Mamaia Pescarie Bay; CTP: Constanta Port; MGP: Mangalia Port.

| Sample | Mean rank | *K1* | *K2* | *df* | *p* | *Pair-wise comparisons* | | | |
|---|---|---|---|---|---|---|---|---|---|
| | | | | | | MPB | CTP | MDP | MGP |
| **MPB** | **46.167** | **18.494** | **7.815** | 3 | 0.0003[1] | | 18.633[1] | 22.700 | 21.333[1] |
| CTP | 27.533 | | | | | −18.633[1] | | 4.067[2] | 2.700[2] |
| MDP | 23.467 | | | | | −22.700[1] | −4.067[2] | | −1.367[2] |
| MGP | 24.833 | | | | | −21.333[1] | −2.700[2] | 1.367[2] | |

[1] Significant difference at $p < 0.05$. [2] Non-significant difference. Bonferroni corrected significance level: 0.0083.
